# Trihalomethanes in Water Supply System and Water Distribution Networks

**DOI:** 10.3390/ijerph18179066

**Published:** 2021-08-27

**Authors:** Sornsiri Sriboonnak, Phacharapol Induvesa, Suraphong Wattanachira, Pharkphum Rakruam, Adisak Siyasukh, Chayakorn Pumas, Aunnop Wongrueng, Eakalak Khan

**Affiliations:** 1 Ph.D.’s Degree Program in Environmental Engineering, Faculty of Engineering, Chiang Mai University, Chiang Mai 50200, Thailand; sornsiri_sri@cmu.ac.th; 2 Graduate School, Chiang Mai University, Chiang Mai 50200, Thailand; 3 Bodhivijjalaya College, Srinakharinwirot University, Nakhon Nayok 26120, Thailand; phacharapol@g.swu.ac.th; 4 Department of Environmental Engineering, Faculty of Engineering, Chiang Mai University, Chiang Mai 50200, Thailand; suraphong@eng.cmu.ac.th (S.W.); pharkphum@eng.cmu.ac.th (P.R.); 5 Department of Industrial Chemistry, Faculty of Science, Chiang Mai University, Chiang Mai 50200, Thailand; adisak.si@cmu.ac.th; 6 Department of Biology, Faculty of Science, Chiang Mai University, Chiang Mai 50200, Thailand; chayakorn.pumas@gmail.com; 7 Research Center in Bioresources for Agriculture, Industry and Medicine, Chiang Mai University, Chiang Mai 50200, Thailand; 8 Research Program in Control of Hazardous Contaminants in Raw Water Resources for Water Scarcity Resilience, Center of Excellence on Hazardous Substance Management, Bangkok 10330, Thailand; 9 Department of Civil and Environmental Engineering and Construction, University of Nevada, Las Vegas, NV 89154, USA; eakalak.khan@unlv.edu

**Keywords:** disinfection byproducts, distribution networks, trihalomethanes

## Abstract

The formation of trihalomethanes (THMs) in natural and treated water from water supply systems is an urgent research area due to the carcinogenic risk they pose. Seasonal effects and pH have captured interest as potential factors affecting THM formation in the water supply and distribution systems. We investigated THM occurrence in the water supply chain, including raw and treated water from water treatment plants (coagulation, sedimentation, sand filtration, ClO_2_-disinfection processes, and distribution pipelines) in the Chiang Mai municipality, particularly the educational institute area. The effects of two seasons, rainy (September–November 2019) and dry (December 2019–February 2020), acted as surrogates for the water quality profile and THM occurrence. The results showed that humic acid was the main aromatic and organic compound in all the water samples. In the raw water sample, we found a correlation between surrogate organic compounds, including SUVA and dissolved organic carbon (DOC) (R^2^ = 0.9878). Four species of THMs were detected, including chloroform, bromodichloromethane, dibromochloromethane, and bromoform. Chloroform was the dominant species among the THMs. The highest concentration of total THMs was 189.52 μg/L. The concentration of THMs tended to increase after chlorination when chlorine dioxide and organic compounds reacted in water. The effect of pH on the formation of TTHMs was also indicated during the study. TTHM concentrations trended lower with a pH ≤ 7 than with a pH ≥ 8 during the sampling periods. Finally, in terms of health concerns, the concentration of TTHMs was considered safe for consumption because it was below the standard (<1.0) of WHO’s Guideline Values (GVs).

## 1. Introduction

Water supply systems are essential to the inhabitants of Chiang Mai Province, Thailand, and the rest of the world. At present, Thailand has a population of 66.19 million people, a growth of 1.01-fold in the past five years [1]. As a result, the demand for water consumption has increased 1.16-fold in this period (from 1835.1 million m^3^ in 2015 to 2121.1 million m^3^ in 2020) [2]. In general, the water supply system consists of coagulation and flocculation, sedimentation, and sand filtration, followed by disinfection, before being pumped through the water distribution networks. The sedimentation and sand filtration processes are highly effective in removing suspended solids and turbidity [3]. However, dissolved organic matter (DOM), which comprises molecules smaller than 0.45 μm, i.e., dissolved organic carbon (DOC), humic acid, and fulvic acid, cannot be effectively removed from the sand filter tank [4]. The most common disinfectants used in the disinfection process are chlorine, chloramines, ozone, and chlorine dioxide [5]. These substances react with DOM, forming disinfectant byproducts (DBPs) that include mutagenic and carcinogenic substances [6,7,8]. There are many types of DBPs, including trihalomethanes (THMs), haloacetic acids (HAAs), and haloacetonitriles (HANs) [9,10]. THMs, including chloroform (CF), bromodichloromethane (BDCM), dibromochloromethane (DBCM), and bromoform (BF), are the dominant DBP species detected in water supply systems [11,12,13]. Consumption of water contaminated with THMs over a long period increases the risk of bladder cancer [14]. Due to adverse health effects caused by DBPs, the World Health Organization (WHO) created guidelines for maximum allowable THMs levels [5]. In addition, the U.S. Environmental Protection Agency (USEPA) states that the maximum contamination level for the four THMs mentioned above is 80 μg/L. In Thailand, water authorities adopted these guidelines to regulate CF, BDCM, DBCM, and BF, with Guideline Values (GVs) of 300, 60, 100, and 100 μg/L, respectively. The sum of the ratios of THMs to their respective GV must be less than one [5]. In addition, iodinated DBPs, such as iodinated THMs, were detected. Ackerson et al. [15] reported that the occurrence of six iodinated THMs (I-THMs; iodoform (TIM), bromodiiodomethane (BDIM), dibromoiodomethane (DBIM), dichloroiodomethane (DCIM), chlorodiiodomethane (CDIM), and chlorobromomethane (CBIM)) was caused by the formation of hypoiodous acid (HOI) during the treatment process. These DBPs are more genetically toxic and cytotoxic than regulated DBPs, including chlorinated and bromine-containing DBPs [16,17]. Many factors have been considered in predicting and controlling DBPs formation, including DOC, ultraviolet absorbance at a wavelength of 254 nm (UV-254), pH, temperature, alkalinity, reaction time, and bromide ions, which strongly impact the distribution of the compounds among the four THM species and may affect reaction yields. Higher THM concentrations generally predict higher levels of the factors mentioned [18,19,20]. The chemical and physical properties of disinfectants and DBPs affect their behavior in drinking water [21,22,23,24]. Ratpukdi et al. [25] showed that high DOC values in water distribution networks from the Khon Kaen Municipality, Thailand, were related to THMs. The highest amounts of THMs and HANs were 584 and 30 μg/L, respectively. Ratpukdi et al.’s study created our concern about DBPs in the water distribution network and water supply system of an educational institute in Chiang Mai Province, Thailand.

In this study, we investigated the seasonal (rainy and dry seasons) concentration of trihalomethanes (THMs) in the water supply system and water distribution networks of an educational institute in Chiang Mai Province, Thailand. The water supply system used chlorine dioxide (ClO_2_) as a disinfectant. The research was performed from September 2019 to February 2020. Other water parameters, including temperature, pH, alkalinity, electrical conductivity, dissolved organic carbon, UV-254 absorbance, and fluorescence excitation–emission matrix (FEEM) measurements, were also conducted.

## 2. Materials and Methods

### 2.1. Water Supply System and Distribution Networks

**Figure 1:**
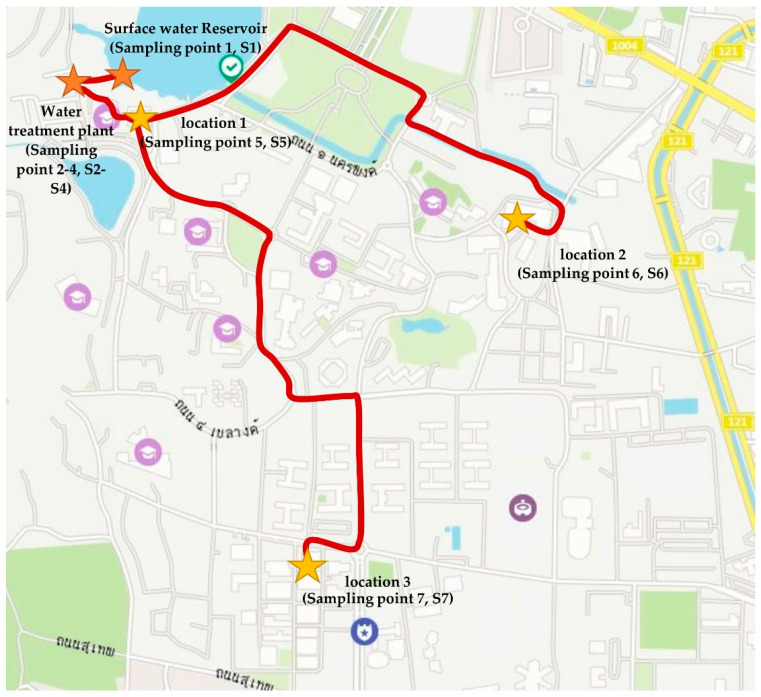
Sampling locations [26].

We examined the water quality of a water supply system located in an educational institute in Chiang Mai, Thailand, that has a capacity of 8000 m^3^/d (Figure 1). The water supply system comprises raw water intake, a coagulation and flocculation unit, sedimentation tank, sand filtration, chlorination tank, and storage tank. The water distribution networks supply water to all buildings and facilities in the educational institute.

### 2.2. Sampling Locations

**Figure 2:**
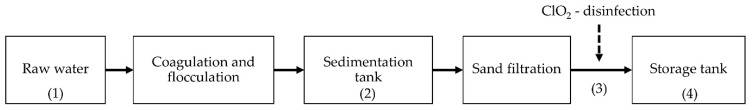
Sampling locations in the water supply system.

Seven sampling points were chosen to assess the water quality of the distribution systems. In the water supply system, four sampling points—raw water (surface water), sedimentation tank, chlorination tank (ClO_2_ disinfection), and storage tank—were selected, as shown in Figure 2. The other three sampling points were spread throughout the distribution lines. Information on sampling location is reported in Table 1.

### 2.3. Sampling Plan

The sampling period was divided into two seasons: rainy (September–November 2019) and dry (December 2019–February 2020). During each collection, five liters of water were sampled. Amber plastic bottles containing the water samples (without headspace) were stored in a dark refrigerator at 4 °C until the analysis.

### 2.4. Analyses

#### 2.4.1. Physical Parameters

The pH level (PH60-E, Apera) and temperature of each water sample were measured at the time of collection. In addition, the samples were analyzed for electrical conductivity (SCHOTT, handy lab LF1).

#### 2.4.2. Chemical Analysis

All samples were pre-filtered using a 0.7 μm GF/F filter followed by a 0.45 μm nylon membrane filter within an hour after collection. The samples were analyzed for CaCO_3_, representing alkalinity (titration method, Standard Method 2320B [27]). During titration, 0.0200 M phosphoric acid was used along with methyl orange as an indicator. The alkalinity equivalent to carbonate was then calculated. DOC was measured by a TOC analyzer (TOC multi N/C 2100, Analytic Jena, Jena, Germany, Standard Method 5310). The ultraviolet absorbance of the sample at 254 nm (UV-254) was also determined using a UV/VIS spectrophotometer (Lambda 365, Perkin Elmer, Perkin Elmer Inc, Boston, MA, USA, Standard Method 5910B). Then, 5.00 g of sodium sulfate anhydrous was added to 25 mL of the sample solution in 40.00 mL amber vials to measure the amount of THMs. The solution was then vigorously stirred to obtain a homogenous solution. After that, 2.5 mL of methyl tertiary butyl ether (MTBE) solution was added and extracted for 3 min. The top layer of MTBE was used for concentration analysis via a gas chromatograph (GC) equipped with an electron capture detector (GC-ECD) system (Agilent 4890 D (EPA 551.1)). The GC column uses a VF-X fused silica capillary column (30m × 0.32 mm × 0.1 µm), and helium was used as a carrier gas [28]. Fluorescence excitation–emission matrices (FEEM) were analyzed to obtain the DOM characteristics of the water samples. The excitation wavelength started from 220 nm to 600 nm and increased by intervals of 5 nm. FEEM spectroscopy was achieved using a spectrofluorometer (JASCO, FP-6200, JASCO international, Tokyo, Japan).

## 3. Results

### 3.1. Physical Parameters

The characteristics of all the samples in the water supply system are shown in Table 2. The water temperature, conductivity, and pH were 25.0–30.7 °C, 130.1–187.7 μS/cm, and 5.9–8.5, respectively.

### 3.2. Chemical Analysis

#### 3.2.1. Alkalinity, DOC, and UV-254 Absorbance

**Figure 3:**
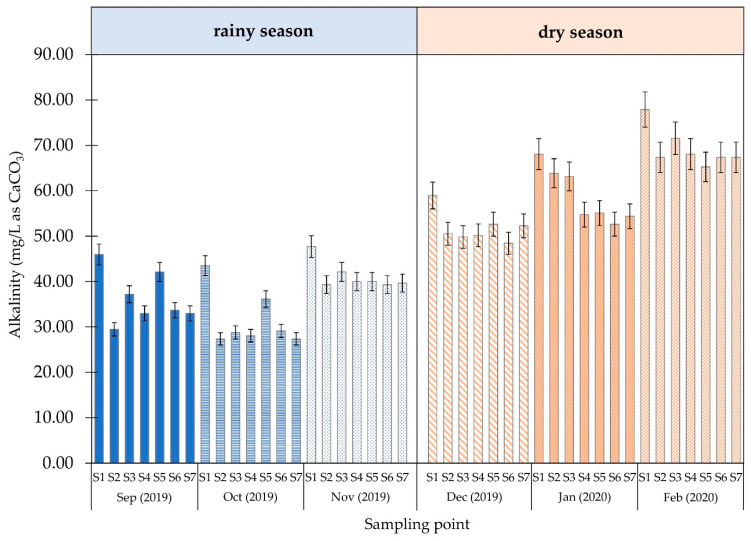
Alkalinity of water at each sampling point.

**Figure 4:**
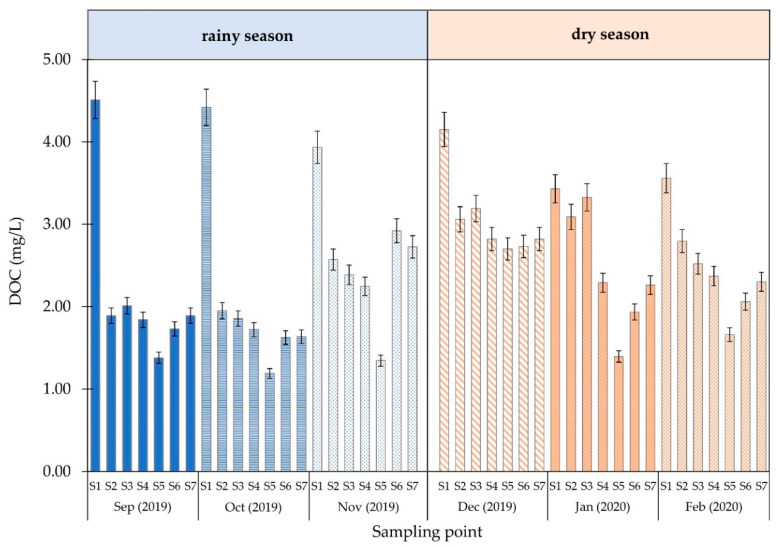
DOC of water at each sampling point.

**Figure 5:**
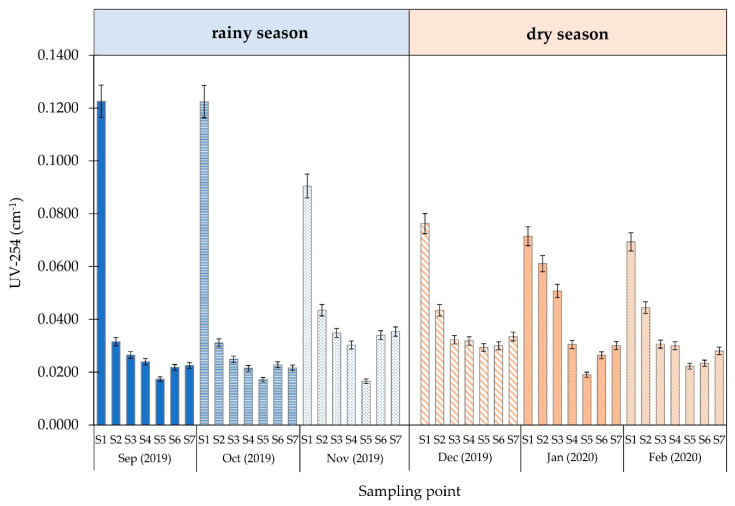
UV absorbance (UV-254) of water at each sampling point.

The results of the alkalinity, DOC, and UV-254 absorbance measurements are shown in Figure 3, Figure 4 and Figure 5 and Table S1 of the Supplementary Materials, respectively.

The alkalinity values (Figure 3) during the rainy season were in the range of 20–40 mg/L of CaCO_3_. However, the value increased in the dry season with a range of 50–75 mg/L of CaCO_3_.

The DOC analysis of the water at various sampling points during the rainy and dry seasons is shown in Figure 4. In both seasons, the result shows that the DOC of the raw water sample from the surface water reservoir was approximately 4.0 mg/L.

The UV-254 absorbance values measured in the raw water sample from the surface water reservoir were approximately 0.120 cm^−1^ and 0.080 cm^−1^ in the rainy and dry seasons, respectively. UV-254 absorbance decreased to approximately 0.030 cm^−1^ in both seasons after passing through water treatment units. The SUVA value of tap water collected each month (rainy and dry seasons) from different locations and the correlation between DOC and SUVA are shown in Table S2 and Figure S1 of the Supplementary Materials, respectively. The SUVA value range for raw water is 1.84–2.77 L/mg·m.

#### 3.2.2. FEEM

**Figure 6:**
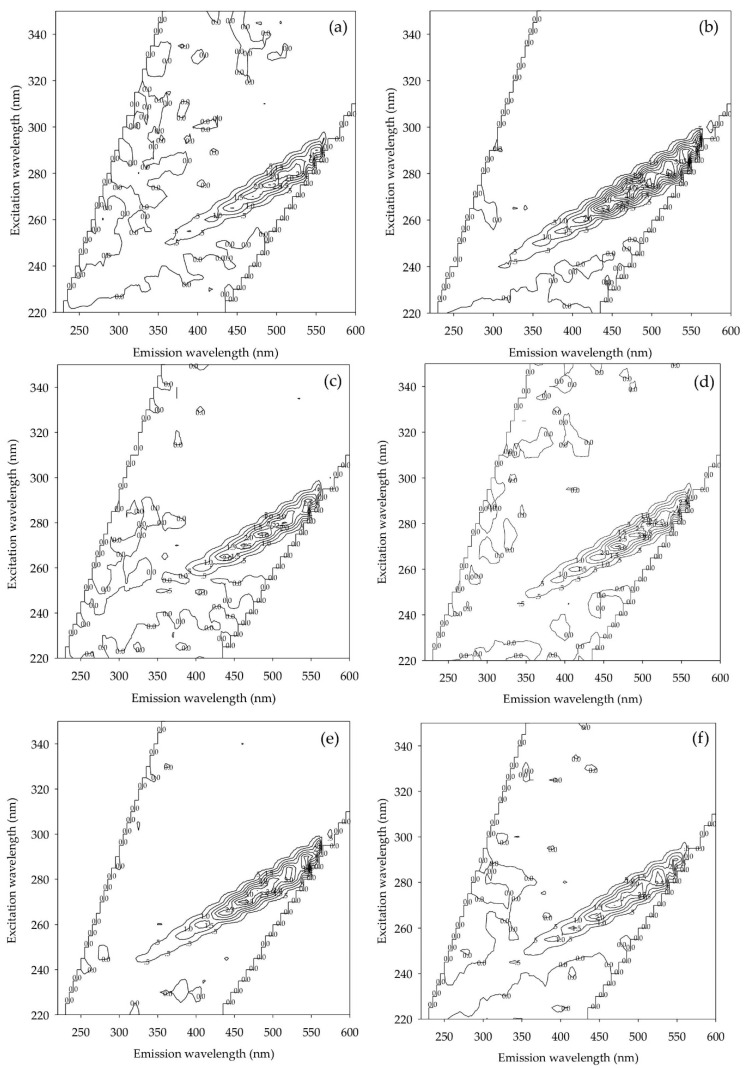
FEEM results of raw water, measured for six months: (**a**) September (2019), (**b**) October (2019), (**c**) November (2019), (**d**) December (2019), (**e**) January (2020), and (**f**) February (2020).

The FEEM technique is a method for identifying natural organic substances, i.e., natural organic matter in water. FEEM peaks at positions (Ex/Em) 270 nm/465 nm, 270 nm/470 nm, 275 nm/490 nm, 270 nm/465 nm, 275 nm/485 nm, and 270 nm/465 nm in September, October, November, December, January, and February, respectively. The FEEM results are shown in Figure 6.

#### 3.2.3. Occurrence of Total Trihalomethanes (TTHMs)

**Figure 7:**
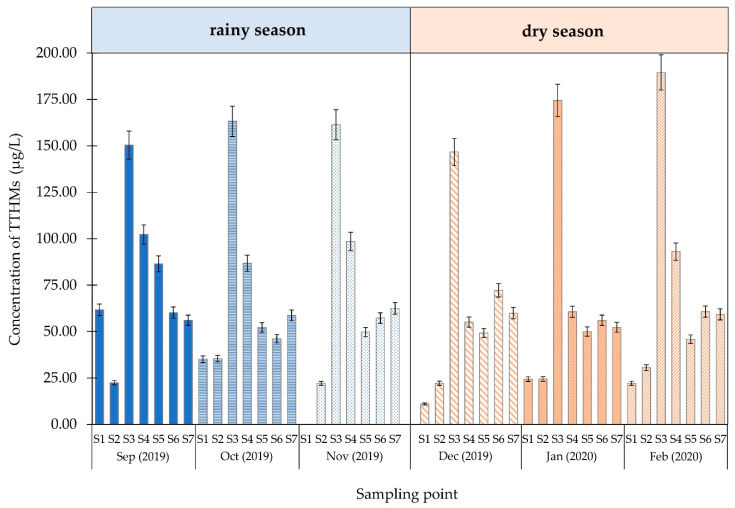
TTHM concentration of water in Chiang Mai, Thailand.

The concentration of TTHMs in the water samples obtained from the sampling locations in Chiang Mai, Thailand, is shown in Figure 7 and the concentration of each THM species is shown in Figure S2 of the Supplementary Materials. The result showed that the water sample from sampling 1 (raw water) and sampling 2 (coagulation process) contained some TTHMs. The highest concentration of TTHMs was 189.52 μg/L at sampling point 3. The sampling points 5–7 were approximately 60.00 μg/L for both seasons.

## 4. Discussion

Regarding the physical parameters of the water profile, the highest pH value was 8.5 for the rainy season and 8.7 for the dry season. The highest temperatures were 30.4 °C and 30.7 °C in the rainy and dry seasons, respectively. However, the conductivity was highest at 287.1 μS/cm in the dry season and 187.7 μS/cm in the rainy season. The water temperature and pH did not vary to any significant degree according to these results. Regardless, the electrical conductivity was lower than the water standard [5].

The highest alkalinity value was 78 mg/L of CaCO_3_ at sampling point 1, measured in February 2020. Compared with the surface water, the water at sampling point 1 underwent sedimentation, resulting in lower alkalinity. The alkalinity decreased from 45 mg/L to 30 mg/L of CaCO_3_ during the rainy season from 70 to 60 mg/L of CaCO_3_ during the dry season. The lower alkalinity value was due to the formation of sediments from using coagulants.

The DOC index analysis was based on the DOC and UV-254 water measurements at each sampling point during the rainy and dry seasons. The increased DOC values for both seasons were comparable to raw water from an alternative tap water source in Thailand. The DOC decreased to approximately 2.0 mg/L in both seasons after passing through the water treatment units. DOC significantly decreased from 9.91% to 58.09 during the rainy season, indicating that the water treatment units removed some organic matter. Monthly changes in the organic content of raw water indicated that the water quality was superior during the rainy season when the organic content was lower. In the rainy season, water turbidity was caused by rain and more organic matter leached from the soil into the stream. Tungsudjawong et al. [29] reported that the Chao Phraya River had more DOC during the rainy season than the dry season. Based on the UV-254 measurement definitions, organic matter containing aromatic clusters and double-bond molecules absorb UV light at a wavelength of 254 nm [30,31]. The obtained UV-254 results showed that the absorbance decreased by approximately 74.63%. In addition, the low absorbance at 254 nm implied that the water from each sampling location contained a small amount of aromatic and double-bond organic compounds. The specific ultraviolet absorption (SUVA) index combines the UV-254 absorbance value and the DOC using a calculation dividing the UV-254 by the DOC and multiplying it by 100. The resulting SUVA value was used to indicate the appropriate type of treatment process for DOMs in water [32]. A SUVA value <2 L/mg·m generally indicates that the sample water contains aliphatic hydrocarbons [33]. ASUVA value >2 mg/L may consist of aromatic hydrocarbon. SUVA value increased during the rainy season compared with the dry season, and the average SUVA (2.60 L/mg·m) was >2 L/mg·m due to the leaching of organic matter into raw water during the rainy season. This was also found for water with a higher ratio of double bonds. This substance may be moderately relevant to THM formation [34].

No significant change occurred in the spectrum peaks of FEEM spectra (Figure 6) for six months. Chen et al. [35] described the extent of excitation (Ex) and emission (Em) wavelengths according to five sections or ″Regions″: In Region 1, Ex/Em values range from 220–250 nm/280–330 nm, representing tyrosine or aromatic proteins. In Region 2, Ex/Em values range from 220–250 nm/330–380 nm, representing BOD_5_ or aromatic proteins. In Region 3, Ex/Em values are 220–250 nm/>380 nm, representing hydrophobic acids or fluvic-acid-like substances. In Region 4, Ex/Em values are >250 nm/280–330 nm and represent soluble microbial byproduct-like substances. Finally, in Region 5, Ex/Em values are >250 nm/>380 nm, representing humic acid-like substances. FEEM within Region 5 had an Ex/Em range of >250/>380 nm for six months, indicating that the natural organic matter in raw water samples could be classified as humic acid. These values are consistent with the SUVA values of raw water containing aromatic organic compounds and suggest that the raw water profile was not affected by seasonal variation.

The TTHMs in the water supply system in Chiang Mai, Thailand, are reported in Figure 7 and the correlation between TTHMs and surrogate parameters (DOC, UV-254 and SUVA) are showed in Figure S3 of the Supplementary Materials. Substantial amounts of THMs were formed after chlorine dioxide disinfection. Four DBP species were detected in the water distribution system, namely, CF, BDCM, DBCM, and BF. The concentration of each THM species for rainy and dry seasons were as follows: CF (0.00–82.09 μg/L), BDCM (0.00–70.62 μg/L), DBCM (0.00–6.83 μg/L), BF (0.00–14.34 μg/L) and CF (10.98–84.27 μg/L), BDCM (0.00–87.12 μg/L), DBCM (0.00–8.71 μg/L), and BF (0.00–14.37 μg/L). CF was the predominant species in this study. Interestingly, the raw water sample obtained from a surface water reservoir contained some DPBs that may have occurred naturally. Since the SUVA value of raw water is >2 L/mg·m, organic compounds are dominated by the double-bond group. These substances, precursors for DBPs (including THMs), are produced by a reaction with chlorine during the disinfection processes. The TTHM concentration was generally lower than the water standard after undergoing the sedimentation process. However, the concentration of TTHMs increased after the chlorination process due to a reaction between ClO_2_ and organic matter. When seasonal effects were compared to the concentration of TTHMs in raw water, we found no significant correlation for either season (*p* > 0.01). When the relevance of average pH in raw water was considered, the average pH of 7.6 during the rainy season was comparable to the average pH of 7.8 during the dry season. Although the average pH for both seasons did not change dramatically, the pH was slightly different at six months, possibly while forming TTHMs. The highest pH during the rainy season was 8.5 in September, followed by 7.8 and 6.6 in October and November, respectively. The TTHM concentrations also increased during these months, with values of 61.75, 35.10, and 0.00 μg/L, respectively. The higher pH level in September may be due to the month’s increased rainfall rate compared with October and November, resulting in enhanced conditions for bacterial and algal growth in the source water. Water containing high amounts of algae and bacteria releases OH− from algal photosynthesis in the water source, causing the pH to increase and create additional TTHMs [36]. Conversely, the dry season’s highest pH levels were in January and February (8.1 and 8.7, respectively), whereas in December, it was 6.6. The high pH in dry seasons may have originated from eutrophication. The TTHM concentrations of 10.98, 24.37, and 22.31 g/L were related to pH values in December, January, and February, respectively. All the water samples in this study contain high values of humic acid; therefore, the formation of THM groups, especially CF, depends on the pH effect. The formation of TTHMs increases when water bodies have a pH of 8 or above [37]. Therefore, TTHMs and pH simultaneously increase when presented with humic substances in raw water. Considering the distance from the water supply point to another point (sampling points 5–7), we found that the concentration of TTHMs in the system decreased as the distance increased due to the degradation of TTHMs. Finally, regarding health concerns, the sum of the ratios of TTHMs from the WHO Guidelines Values (GVs) was greater than one at the distribution point (S5–S7), indicating that the water supply was safe for consumption.

## 5. Conclusions

In this study, we tested the efficiency of the water supply system in an educational institute located in Chiang Mai Province, Thailand. Our results showed that the FEEM values were in the range of >250/>380 nm and the SUVA value was >2 L/mg·m. We assumed that the raw water sample had aromatic hydrocarbons (humic acid) from organic matter. In addition, the DOC value decreased by 58.09%. The UV-254 absorbance decreased the most, by 74.63%. Four types of THMs were found after adding disinfectants during the disinfection process: CF, BDCM, DBCM, and BF. CF was the predominant species in this study. Additionally, TTHMs discovered in the water at sampling point 1 (surface water) may have occurred naturally. We found a decrease in the amount of TTHMs as the water underwent coagulation and flocculation processes. We observed that seasonal changes did not affect the incidence of TTHMs in raw water, whereas pH plays an important role in determining the type and amount of DBPs formed. Overall, the treated water is safe for consumption following the Guideline Values (GVs < 1.0).

## Figures and Tables

**Table 1:** Information on sampling locations.

Distribution System	Sampling Point	Distance from Water Treatment Plant (km)	Coordinates	Information on Sampling Location
surface water reservoir	S1	0.20	18°48′23.4″ N 98°56′59.0″ E	surface water
water treatment plant	S2	0.00	18°48′19.5″ N 98°56′49.6″ E	sedimentation tank
water treatment plant	S3	0.00	18°48′19.5″ N 98°56′49.6″ E	chlorination tank
water treatment plant	S4	0.10	18°48′19.5″ N 98°56′49.6″ E	after chlorination tank
location 1	S5	0.13	18°48′17.2″ N 98°56′53.4″ E	-
location 2	S6	1.00	18°48′10.8″ N 98°57′25.0″ E	-
location 3	S7	1.13	18°47′45.3″ N 98°57′03.8″ E	-

**Table 2:** Water temperature, conductivity, and pH of water samples.

Parameters	WHO Acceptable Value [5]	Season/Month		Sampling Point						
				S1	S2	S3	S4	S5	S6	S7
pH	6.5–8.5	rainy	Sep	8.5	7.1	6.8	7.5	7.5	7.5	7.4
			Oct	7.8	6.9	5.9	7.1	7.4	6.5	7.6
			Nov	6.6	6.5	6.1	7.0	6.3	6.5	6.5
		dry	Dec	6.6	6.3	6.1	6.7	6.5	6.5	6.6
			Jan	8.1	7.8	7.7	7.4	7.2	7.1	7.1
			Feb	8.7	7.4	7.5	7.3	7.3	7.2	7.3
Water temperature (°C)	-	rainy	Sep	30.7	28.7	29.0	38.7	28.0	28.0	28.0
			Oct	30.0	28.5	28.2	26.3	28.0	28.5	28.0
			Nov	29.7	28.2	27.8	28.0	26.1	26.5	25.0
		dry	Dec	30.0	29.0	28.0	28.0	27.0	26.5	26.1
			Jan	30.2	29.8	29.5	29.3	28.7	28.9	29.0
			Feb	30.4	30.0	29.4	29.1	29.1	28.9	28.9
Electroconductivity (μS/cm)	<500	rainy	Sep	130.1	149.8	186.7	154.5	157.7	165.0	155.9
			Oct	141.2	161.9	183.4	166.5	187.7	167.5	168.6
			Nov	131.4	155.2	165.2	149.8	161.1	147.2	149.8
		dry	Dec	181.7	193.9	287.1	274.9	259.0	230.8	254.1
			Jan	170.0	172.7	188.8	194.7	184.1	185.7	186.9
			Feb	174.9	190.6	208.3	197.8	212.5	200.4	191.0

## Data Availability

Not applicable.

## Supplementary Materials

The following are available online at https://www.mdpi.com/article/10.3390/ijerph18179066/s1, Table S1: Water quality parameters at sampling point, Table S2: Water quality parameters at sampling point, Figure S1, Correlation between UV-254 and DOC (a) and SUVA and DOC (b) in raw water (surface water), Figure S2: The concentration of THMs species (μg/L); (a) Concentration of CF, (b) Concentration of BDCM, (c) Concentration of DBCM and (d) Concentration of BF., and Figure S3. Correlation between concentration of TTHMs and DOC (a), concentration of TTHMs and UV-254 (b), and concentration of TTHMs and SUVA in raw water (surface water).
