# Supplementary material for: Trihalomethanes in Water Supply System and Water Distribution Networks
Source: Int J Environ Res Public Health. 2021 Aug 27;18(17):9066. doi: 10.3390/ijerph18179066 (PMC8430772; doi:10.3390/ijerph18179066)
Supplement: Supplementary file 1 [file ijerph-18-09066-s001.zip › ijerph-1329844-supplementary.pdf]

Supplementary Materials

Trihalomethanes in Water Supply System and Water Distribution Networks

Sornsiri Sriboonnak [1,2], Phacharapol Induvesa [3], Suraphong Wattanachira [4], Pharkphum Rakruam [4], Adisak Siyasukh [5], Chayakorn Pumas [6,7], Aunnop Wongrueng [4,7,8,*] and Eakalak Khan [9]

[1] Ph.D.'s Degree Program in Environmental Engineering, Faculty of Engineering, Chiang Mai University, Chiang Mai 50200, Thailand; sornsiri_sri@cmu.ac.th
[2] Graduate School, Chiang Mai University, Chiang Mai 50200, Thailand
[3] Bodhivijjalaya College, Srinakharinwirot University, Nakhon Nayok 26120, Thailand; phacharapol@g.swu.ac.th
[4] Department of Environmental Engineering, Faculty of Engineering, Chiang Mai University, Chiang Mai 50200, Thailand; suraphong@eng.cmu.ac.th (S.W.); pharkphum@eng.cmu.ac.th (P.R.); aunnop@eng.cmu.ac.th (A.W.)
[5] Department of Industrial Chemistry, Faculty of Science, Chiang Mai University, Chiang Mai 50200, Thailand; adisak.si@cmu.ac.th
[6] Department of Biology, Faculty of Science, Chiang Mai University, Chiang Mai 50200, Thailand; chayakorn.pumas@gmail.com
[7] Research Center in Bioresources for Agriculture, Industry and Medicine, Chiang Mai University, Chiang Mai 50200, Thailand
[8] Research Program in Control of Hazardous Contaminants in Raw Water Resources for Water Scarcity Resilience, Center of Excellence on Hazardous Substance Management, Bangkok 10330, Thailand
[9] Department of Civil and Environmental Engineering and Construction, University of Nevada, Las Vegas, NV 89154, USA; eakalak.khan@unlv.edu
* Correspondence: aunnop@eng.cmu.ac.th; Tel.: +66-53-94-4101-3

Table S1. Water quality parameters at sampling point.

Parameters	Season/Month		Sampling Point						
			S1	S2	S3	S4	S5	S6	S7
Alkalinity (mg/L of $CaCO_3$)	wet	Sep	45.96±1.16	29.47±3.65	37.19±1.22	32.98±1.22	42.10±0.00	33.68±1.05	32.98±1.22
		Oct	43.50±1.22	27.37±0.00	28.77±1.22	28.07±1.22	36.14±0.61	29.12±0.61	27.37±0.00
		Nov	47.71±0.61	39.29±1.22	42.10±1.05	40.00±0.00	40.00±0.00	39.29±0.61	39.64±0.61
	dry	Dec	58.94±0.00	50.52±0.00	49.82±1.22	50.17±0.61	52.63±0.00	48.42±0.00	52.27±1.61
		Jan	68.06±1.22	63.85±1.22	63.15±0.00	54.73±0.00	55.08±0.61	52.63±0.00	54.38±0.61
		Feb	77.89±0.00	67.36±0.00	71.57±0.00	68.06±1.22	65.26±0.00	67.36±0.00	67.36±0.00
DOC (mg/L)	wet	Sep	4.51±0.03	1.89±0.04	2.01±0.04	1.84±0.00	1.38±0.01	1.73±0.01	1.89±0.04
		Oct	4.42±0.03	1.95±0.00	1.86±0.08	1.72±0.04	1.19±0.03	1.63±0.02	1.63±0.01
		Nov	3.94±0.04	2.57±0.03	2.39±0.09	2.25±0.01	1.35±0.01	2.92±0.01	2.73±0.06
	dry	Dec	4.16±0.01	3.06±0.07	3.19±0.04	2.82±0.06	2.71±0.01	2.73±0.03	2.82±0.01
		Jan	3.43±0.03	3.09±0.04	3.33±0.01	2.29±0.08	1.40±0.01	1.94±0.05	2.26±0.03
		Feb	3.56±0.01	2.80±0.01	2.52±0.01	2.37±0.01	1.66±0.04	2.06±0.03	2.30±0.06
UV-254 (cm^{-1})	wet	Sep	0.1226±0.0001	0.0315±0.0001	0.0264±0.0001	0.0239±0.0002	0.0173±0.0001	0.0218±0.0001	0.0225±0.0000
		Oct	0.1225±0.0001	0.0311±0.0001	0.0249±0.0001	0.0214±0.0001	0.0171±0.0001	0.0228±0.0001	0.0216±0.0001
		Nov	0.0905±0.0001	0.0435±0.0001	0.0348±0.0001	0.0303±0.0001	0.0166±0.0001	0.0340±0.0001	0.0354±0.0001
	dry	Dec	0.0763±0.0001	0.0434±0.0001	0.0323±0.0001	0.0318±0.0001	0.0293±0.0001	0.0230±0.0001	0.0335±0.0001
		Jan	0.0715±0.0001	0.0611±0.0000	0.0507±0.0001	0.0305±0.0001	0.0190±0.0001	0.0264±0.0001	0.0301±0.0001
		Feb	0.0693±0.0001	0.0444±0.0001	0.0306±0.0001	0.0300±0.0000	0.0223±0.0001	0.0234±0.0001	0.0280±0.0001

Table S2. Water quality parameters at sampling point.

Parameters	Season/Month		Sampling Point						
			**S1**	**S2**	**S3**	**S4**	**S5**	**S6**	**S7**
SUVA (L/mg·m)	wet	Sep	2.72	1.67	1.32	1.30	1.26	1.26	1.19
		Oct	2.77	1.59	1.34	1.25	1.44	1.40	1.32
		Nov	2.30	1.69	1.46	1.35	1.23	1.16	1.30
	dry	Dec	1.84	1.42	1.01	1.13	1.09	1.10	1.19
		Jan	2.08	1.98	1.53	1.33	1.36	1.36	1.33
		Feb	1.95	1.59	1.22	1.27	1.34	1.13	1.22
G.V.	wet	Sep	0.46	0.22	1.44	0.90	0.54	0.61	0.61
		Oct	0.35	0.35	1.56	0.82	0.43	0.40	0.48
		Nov	0.00	0.22	1.46	1.02	0.42	0.46	0.52
	dry	Dec	0.04	0.22	1.58	0.54	0.42	0.59	0.54
		Jan	0.12	0.22	1.84	0.56	0.38	0.45	0.48
		Feb	0.22	0.30	1.88	0.94	0.41	0.49	0.52

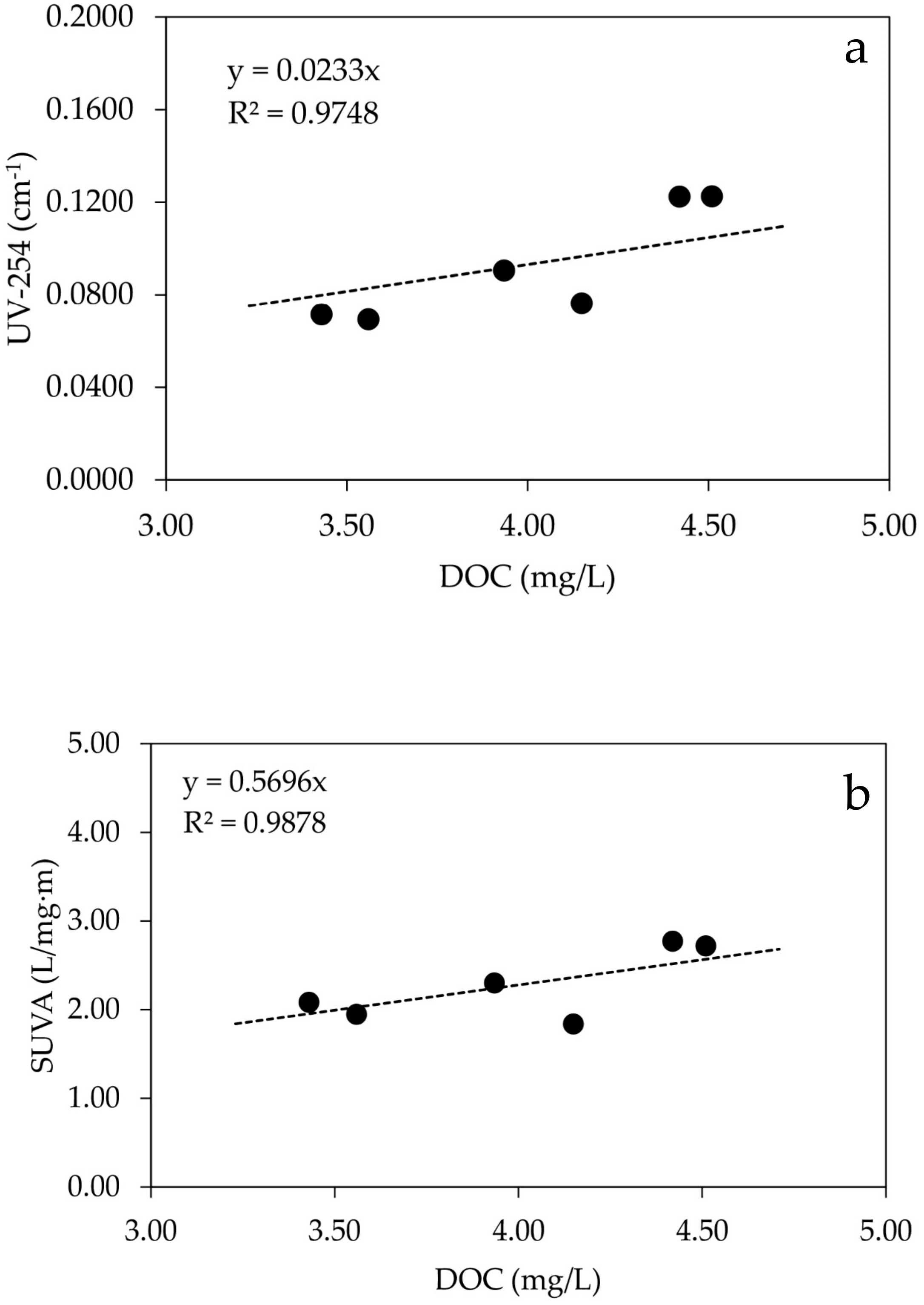

Figure S1. Correlation between UV-254 and DOC (**a**) and SUVA and DOC (**b**) in raw water (surface water).

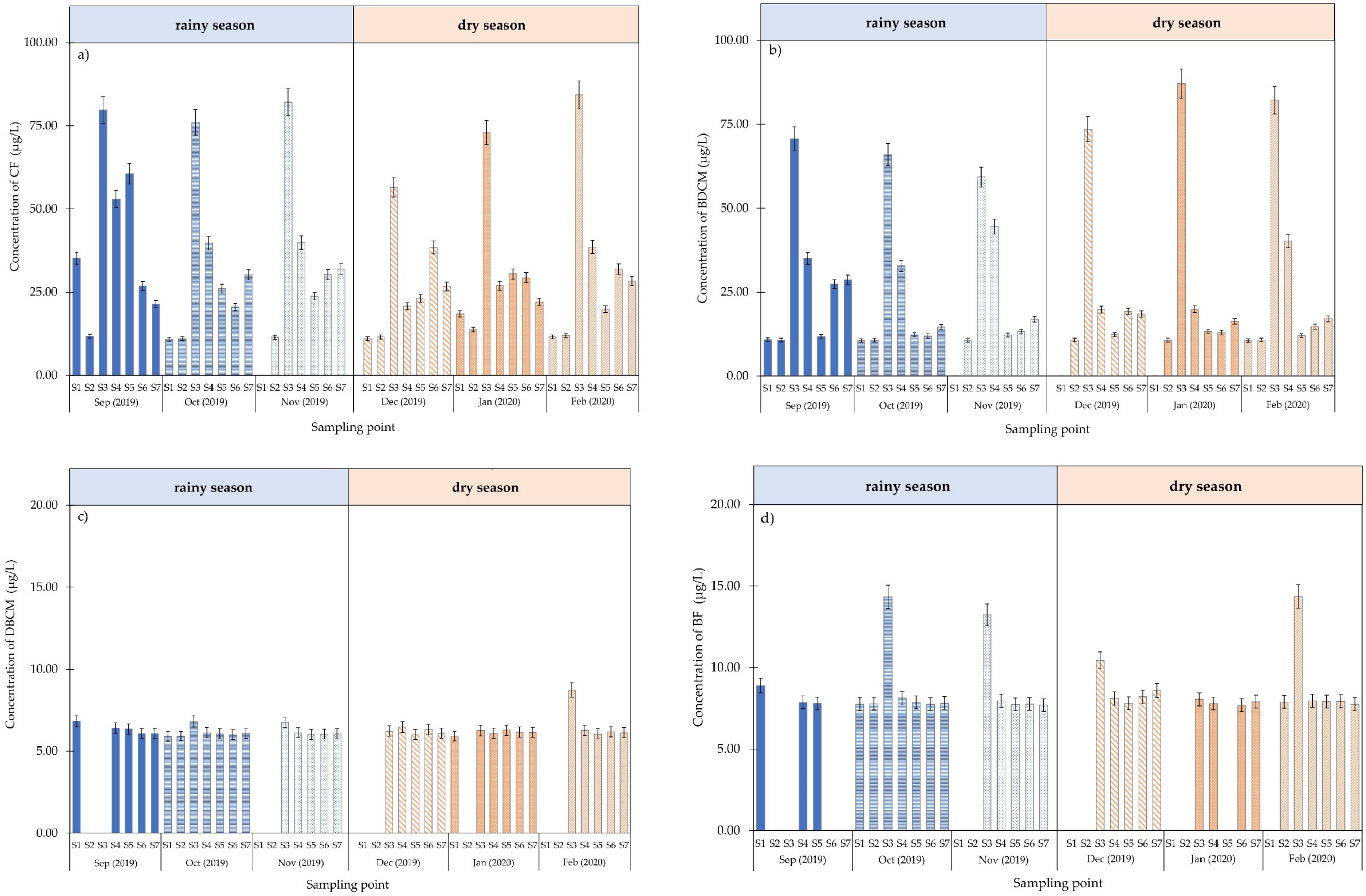

Figure S2. The concentration of THMs species (μg/L); (**a**) Concentration of CF, (**b**) Concentration of BDCM, (**c**) Concentration of DBCM and (**d**) Concentration of BF.

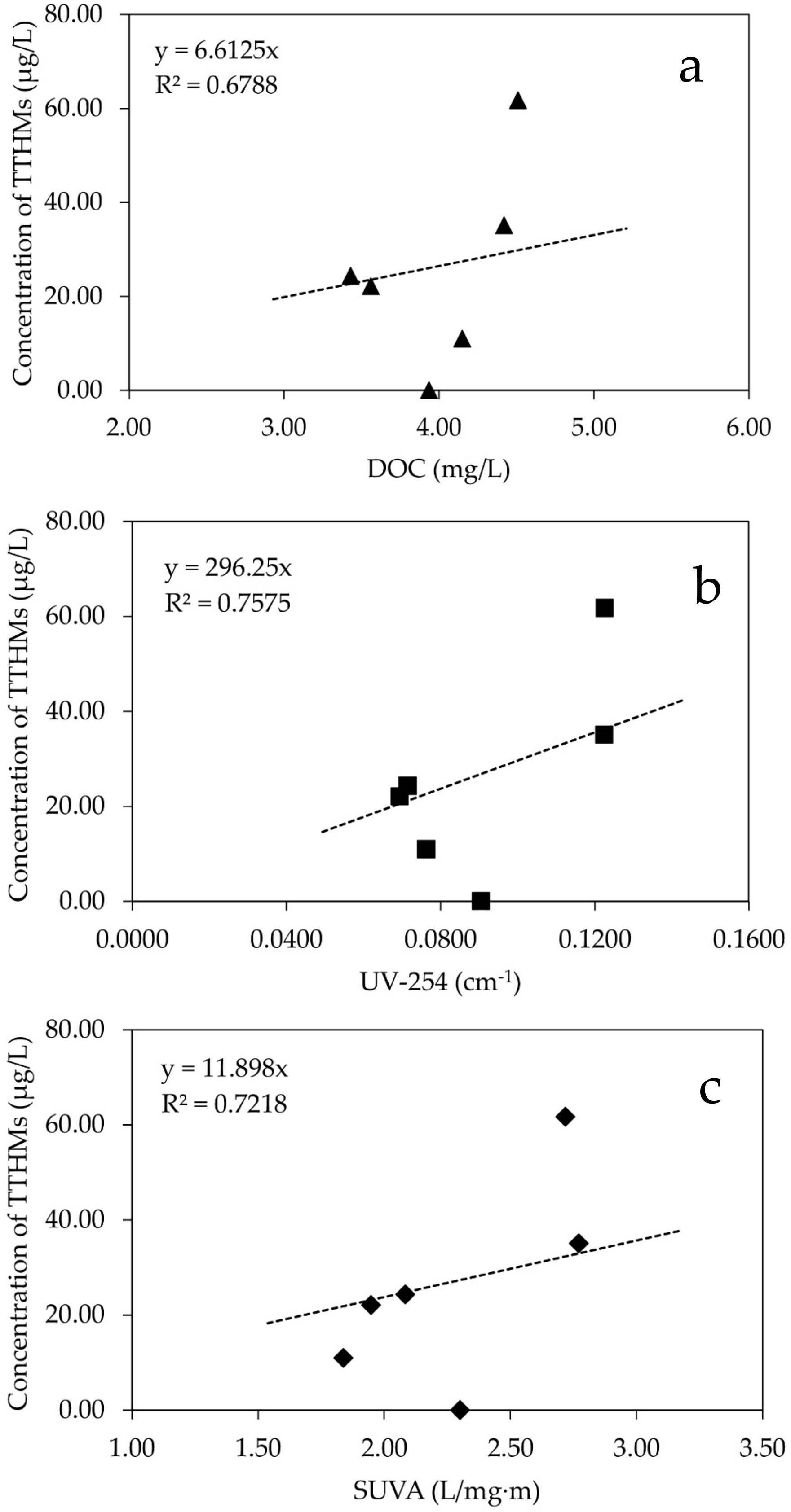

Figure S3. Correlation between concentration of TTHMs and DOC (**a**), concentration of TTHMs and UV-254 (**b**) and concentration of TTHMs and SUVA(**c**) in raw water (surface water.
